# Rift Valley fever virus NSs protein functions and the similarity to other bunyavirus NSs proteins

**DOI:** 10.1186/s12985-016-0573-8

**Published:** 2016-07-02

**Authors:** Hoai J. Ly, Tetsuro Ikegami

**Affiliations:** Department of Pathology, The University of Texas Medical Branch at Galveston, Galveston, TX USA; The Sealy Center for Vaccine Development, The University of Texas Medical Branch at Galveston, Galveston, TX USA; The Center for Biodefense and Emerging Infectious Diseases, The University of Texas Medical Branch at Galveston, Galveston, TX USA

**Keywords:** Rift Valley fever virus, Phlebovirus, Bunyavirus, NSs, PKR, TFIIH, p62, p53, Ubiquitin, Interferon, E3 ligase

## Abstract

Rift Valley fever is a mosquito-borne zoonotic disease that affects both ruminants and humans. The nonstructural (NS) protein, which is a major virulence factor for Rift Valley fever virus (RVFV), is encoded on the S-segment. Through the cullin 1-Skp1-Fbox E3 ligase complex, the NSs protein promotes the degradation of at least two host proteins, the TFIIH p62 and the PKR proteins. NSs protein bridges the Fbox protein with subsequent substrates, and facilitates the transfer of ubiquitin. The SAP30-YY1 complex also bridges the NSs protein with chromatin DNA, affecting cohesion and segregation of chromatin DNA as well as the activation of interferon-β promoter. The presence of NSs filaments in the nucleus induces DNA damage responses and causes cell-cycle arrest, p53 activation, and apoptosis. Despite the fact that NSs proteins have poor amino acid similarity among bunyaviruses, the strategy utilized to hijack host cells are similar. This review will provide and summarize an update of recent findings pertaining to the biological functions of the NSs protein of RVFV as well as the differences from those of other bunyaviruses.

## Background

Rift Valley fever (RVF) is a zoonotic viral disease transmitted by mosquitoes. It was first identified in Kenya in 1930 [1], and has been endemic in sub-Saharan Africa for more than 80 years [2]. Outbreaks of RVF have also occurred in Madagascar, Egypt, Saudi Arabia, and Yemen, probably through infected animals or mosquitoes [2–6]. The largest recorded RVF outbreak occurred in Egypt back in 1977–78, where there were an estimate of 20,000 to 200,000 human cases and roughly 600 confirmed deaths [5]. The disease is caused by the Rift Valley fever virus (RVFV), which belongs to the genus *Phlebovirus* within the family *Bunyaviridae* [7]. Pregnant cattle, goats, and especially sheep, are highly susceptible to RVFV, resulting in fetal malformation (e.g., hydrop amnii, arthrogryposis, scoliosis, hydraencephaly, cerebellar hypoplasia) and abrupt abortion in 40 to 100 % of pregnant ewes [2, 8]. Newborn lambs are also highly susceptible to RVFV, and the mortality rate is estimated to be 95 to 100 % [2]. In adult ruminants, transient fever and viremia are generally displayed and the mortality rate of infected sheep has been documented to be up to 20 % [9]. Most human cases are characterized with self-limiting febrile illness, and less than 8 % of patients develop a more severe form of the disease, such as hemorrhagic fever, encephalitis, or retinitis. The overall mortality rate for RVF patients is 0.5 to 1 % [10–12]. Through vertical transmission, flood water *Aedes* mosquitoes play an important role in the maintenance of RVFV in nature [13, 14]. Periods of heavy rainfall generates ideal conditions for the hatching of infected *Aedes* eggs [13], thus triggering the transmission of RVFV to animals. Transmission of the disease to humans (e.g., veterinarian, farmers, or abattoir workers) can then be accomplished through direct contact with bodily fluids derived from infected animals. However, during an outbreak, infected mosquito vectors, such as *Culex pipiens,* could also play an important role in RVFV transmission to humans [14, 15]. Potential spread of RVFV, either naturally or intentionally, into non-endemic countries is a major public health concern. Due to a concern towards bioterrorism in the U.S., RVFV is classified as Category A Priority Pathogen (NIAID/NIH), and an overlap select agent (U.S. Department of Human Health Services, and U.S. Department of Agriculture). A strict quarantine system is important to prevent the importation of RVFV infected animals [16]. However, the case is not the same for humans, because infected patients can travel by airplanes, and may bring RVFV from endemic countries into non-endemic area [17]. Fortunately, human to human transmission has not been documented and uninfected individuals are unlikely able to trigger RVF outbreaks. Currently, there are no licensed RVF vaccines for humans, and only a few licensed vaccines are available for animals [18], such as the live-attenuated MP-12 vaccine, which is conditionally licensed in the U.S. [19]. No effective therapeutic is available for RVF patients. Reduction of viral load with increased survival rate was shown in mice or nonhuman primates infected with RVFV through daily post-exposure treatment with ribavirin (a guanosine analog), Poly (ICLC) (a polyriboinosinic-polyribocytidylic acid stabilized with poly-L-lysine and carboxymethyl cellulose), recombinant IFN-αA or human IFN-α [20–25]. However, both ribavirin and IFN-α have side effects associated with therapeutic use [26]. Improvement of antivirals that is not only effective for RVFV, but for other pathogenic bunyaviruses, could be important countermeasures against the threat that bunyaviruses impose. Currently, the challenge lies within the development of broadly-active antivirals or vaccines that can encompass the large genetic diversity of bunyaviruses.

The bunyavirus genome is comprised of three negative-sense single stranded RNA segments; Large (L)-, Medium (M)-, and Small (S)-segments. The L-segment encodes the RNA-dependent RNA polymerase (L protein). The M-segment encodes a single open reading frame (ORF) for glycoprotein precursor gene that produces the envelope glycoproteins; Gn and Gc. The S-segment encodes for the nucleoprotein (N protein). A nonstructural protein, NSm, is encoded in the M-segment of orthobunyaviruses, nairoviruses, tospoviruses, or sandfly or mosquito-borne phleboviruses, while NSs protein is encoded in the S-segment of orthobunyaviruses, phlebovirues, nairovirus, or hantaviruses that are transmitted by *Arvicolinae* or *Sigmodontinae* rodents, but not by *murinae* rodents [7, 27–31].

Termini on the viral genome RNA are complementary and forms a pan-handle structure, which serves as signals for N protein encapsidation and RNA synthesis [7]. Bunyavirus L proteins cleave capped host mRNA to initiate the synthesis of viral mRNA [32–35]. This cap-snatching mechanism is also commonly used in other segmented negative-stranded RNA viruses (e.g., influenza viruses, and arenaviruses). The transcription of bunyavirus mRNA is terminated within the untranslated region (UTR) [36–40], while L protein do not recognize the termination signal during the mode of viral genomic RNA synthesis. The mechanism of transcription and genome replication, however, remains largely unknown.

RVFV M-segment encodes two precursor proteins, 78kD-Gc and NSm-Gn-Gc, for the expression of 78kD, NSm, Gn, and Gc proteins through co-translational cleavage of precursor protein via signal peptidases [41]. The 78kD protein is a structural protein that is incorporated into virions matured from mosquito cells, but not those from Vero cells [42]. The 78kD protein is important for efficient viral dissemination in mosquitoes [43–45], while the NSm protein localizes to the mitochondrial outer membrane, and delays apoptosis in mammalian cells [46, 47].

## Genetic diversity among bunyaviruses

**Fig. 1:**
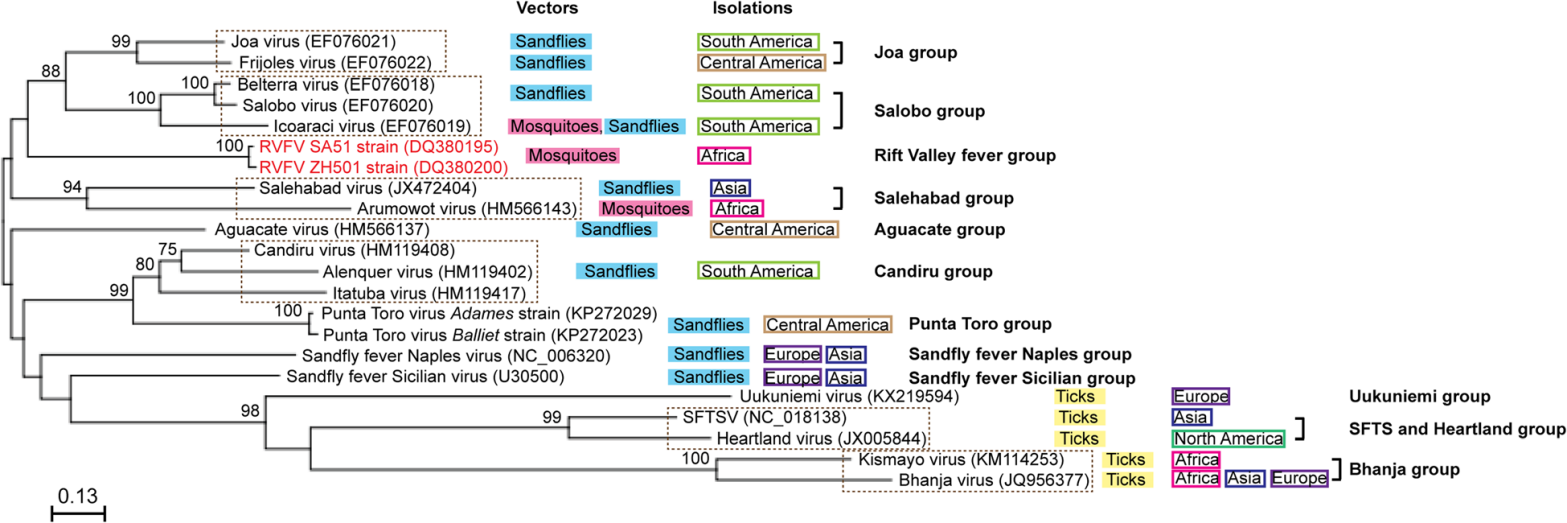
Genetic diversity of phleboviruses. Phylogenetic analysis of available phlebovirus M-segment partial ORF (corresponding to RVFV M precursor polyprotein amino acid position 583 to 770) at amino acid level was performed. The neighbor-joining method with Kimura two-parameter distance was utilized to generate phylogenetic tree with 1000 replicates, using CLC Genomics Workbench version 7.5.2. Vectors and isolations of each virus are also shown. Relevant studies have been reported previously [51, 57, 141–148]

The *Bunyaviridae* family consists of more than 350 distinct viruses, and belongs to the genus *Orthobunyavirus*, *Phlebovirus*, *Nairovirus*, *Hantavirus*, and *Tospovirus* [7]. Phleboviruses consists of at least 70 named viruses, and is divided into two antigenic groups; the sandfly fever group, and the Uukuniemi group, which is based on the presence of the preglycoprotein coding region in the M-segment [48]. Traditionally phlebovirus species were defined by the distinct antigenic cross-reactivity, however, some phleboviruses do not form clear plaques in mammalian cells, and the antigenic cross-reactivity has not been well characterized. A number of viruses have not been approved as phlebovirus species partly due to such technical inconvenience. Phleboviruses are currently comprised of the following genetically distinct groups (Fig. 1); (i) sandfly or mosquito-borne phleboviruses; Rift Valley fever group, Sandfly fever Naples group (e.g., Toscana virus: TOSV), Sandfly fever Sicilian group (e.g., Sandfly fever Sicilian virus: SFSV), Joa group (e.g., Frijoles virus: FRIV), Salobo group (e.g., Icoaraci virus: ICOV, Belterra virus), Candiru group (e.g., Candiru virus, Alenquer virus, Itaituba virus), Punta Toro group (e.g., Punta Toro virus: PTV), Salehabad group (e.g., Salehabad virus, Arumowot virus), and Aguacate group (e.g., Aguacate virus), (ii) tick-borne phleboviruses; Uukuniemi group (e.g., Uukuniemi virus: UUKV), Bhanja group (e.g., Bhanja virus, Kismayo virus), Kaisodi group (e.g., Kaisodi virus, Lanjan virus), and Severe Fever with Thrombocytopenia Syndrome (SFTS) and Heartland group (e.g., SFTSV, Heartland virus). Sandfly fever is characterized by a self-limiting febrile illness in humans, whereas TOSV is known to cause aseptic meningitis or meningoencephalitis [49, 50]. Most sandfly fever viruses are transmitted by sandfly species within genus *Phlebotomus* (old world sandflies: Sandfly fever Naples group, Sandfly fever Sicilian group, and Salehabad group) or genus *Lutzomya* (new world sandflies: Punta Toro group, Joa group, Salobo group, Aguacate group, Joa group, and Candiru group). On the other hand, RVFV, ICOV, Arumowot virus, and Itaporanga virus (this virus still remains to be grouped) transmitted by mosquitoes [51]. SFTSV is transmitted by ticks (*Haemaphysalis longicornis* or *Rhipicephalus microplus*) in China, and the disease is characterized by an acute onset of fever, accompanied by thrombocytopenia, leukopenia, and diarrhea in humans, with the mortality rate of 12 to 30 % [52, 53]. Patients with SFTSV were also identified in Korea and Japan [52–55]. Heartland virus is transmitted by lone star ticks (*Amblymma americanum*) in the U.S., and the disease is characterized with the onset of fevers with thrombocytopenia and leukopenia [56].

The NSs protein is considered to be the major virulence factor for bunyaviruses, and plays an important role in viral evasion from the host innate immunity. It is important to characterize common biological functions of bunyavirus NSs proteins toward the development of effective therapeutic regimens and vaccines. However, NSs proteins exhibit a wide diversity of amino acid sequences among sandfly fever phleboviruses [57, 58]. For example, the amino acid identities of NSs proteins among RVFV, SFSV, TOSV, PTV, ICOV, and FRIV are low, and range from 7.5 to 28.6 % [57]. Although phleboviruses are genetically diverse, identification of common mechanisms utilized by phlebovirus NSs proteins may lead to the development of broadly-active antiviral molecules. We will describe the current understanding of RVFV NSs functions, as well as discuss the similarities and differences observed in NSs proteins of other bunyaviruses in following sections.

## Inhibition of host general transcription by bunyavirus NSs proteins

RVFV NSs proteins are present in both the nucleus and cytoplasm of infected cells, and it forms a filamentous structure in nucleus [59]. The NSs protein of RVFV is comprised of 265 amino acids (aa.) and is estimated to be 31kD in size [7]. The 17aa. at the C-terminus is highly acidic, and is responsible for the formation of the filamentous structure seen in the nucleus [59]. Currently, no nuclear localization signal for RVFV NSs protein has been identified, however, nuclear accumulation of RVFV NSs protein has been shown to be dependent on PXXP motifs (P: proline, X: any amino acid) [60]. RVFV NSs protein also encodes the FVEV (ΩXaV motif: Ω, omega: Trp or Phe, X: any amino acid, a: Asp or Glu, V: Val) within the last 17 amino acids of the C-terminus. Recombinant RVFV encoding NSs lacking the ΩXaV motif, failed to form NSs filaments in the nucleus, indicating the role of ΩXaV motif in filament formation [61].

Le May et al. demonstrated that RVFV NSs protein inhibits cellular general transcription activity by directly interacting with transcription factor (TF) IIH components [62]. The TFIIH is comprised of ten subunits; i.e., (i) the core complex: XPB, XPD, p52, p44, p62, p34, and p8, and (ii) the cyclin activating kinase sub-complexes: cdk7, cyclin H, and MAT1. As one of the basal transcription factors, TFIIH along with TFIIA, TFIIB, TFIID, TFIIE, and TFIIF, forms the transcription preinitiation complex for RNA polymerase II. TFIIH is involved in promoter melting and allowing Pol-II to escape from the promoter to transition from the initiation complex to the elongation complex via cyclin-dependent phosphorylation of the Pol-II large subunit C-terminal domain (CTD). TFIIH is also required for RNA polymerase I-mediated transcription [63]. Synthesis of both poly-A(+) and poly-A(−) RNA was decreased in the presence of RVFV NSs protein. The XPB and p44 subunit was shown to co-localize with RVFV NSs filaments, unlike p62, p52, cdk7, and XPD, which did not. It was also observed that XPD and p62 proteins were decreased in the presence of NSs proteins, whereas the p44, XPB, and TBP (TATA-binding protein) did not. NSs proteins bound to p44, but not directly to XPB. The NSs protein did not interact with p44 subunits that had been already incorporated into the TFIIH complex. From what had been gathered, it was hypothesized that RVFV NSs protein competes for the binding between XPD and p44, thus preventing the assembly of the TFIIH complex in the nucleus.

In later studies, Kalveram et al. demonstrated that RVFV NSs protein interacts with TFIIH p62, and subsequently promotes its post-translational degradation [64]. The p62 protein was stabilized in the presence of proteasome inhibitors, MG132 or lactacystin, and degradation occurred even in the presence of a nuclear export inhibitor, leptomycin B, or a lysosomal inhibitor, chloroquine. These studies demonstrated that RVFV NSs protein induces host transcriptional shutoff, by two potentially redundant mechanisms: (1) interruption of TFIIH complex assembly by NSs-p44 binding, and (2) post-translational degradation of TFIIH p62 subunit.

Cellular protein degradation occurs mainly through the ubiquitin (Ub)-proteasome system, where polyubiquitination of proteins with chains of at least four Lys 48-linked Ubs leads to degradation through the 26S proteasome [65, 66]. To ubiquitinate proteins, three sequential steps are necessary; (i) Ub-binding to E1 (the Ub activating enzyme), (ii) transfer of Ub to E2 (the Ub conjugating enzyme) and (iii) transfer of Ub from E2 to a substrate protein in the E3 ligase complex [66–68]. To promote the transfer of Ub to TFIIH p62, it was hypothesized that RVFV NSs protein serves as an adaptor protein in the E3 ligase complex [69]. The human cell encodes 8 different cullins (CUL1, 2, 3, 4A, 4B, 5, 7, and 9) [70]. As a scaffold protein, cullin bridges either the Rbx1 or Rbx2 proteins, which encodes for the RING domain. The ligase activity of the E3 ligase complex is dependent on the covalent modification of cullin with the ubiquitin-like modifier NEDD8 [71]. Different cullins bind to their respective adaptor proteins; e.g., CUL1 or CUL7 binds to Skp1 protein, CUL2 or CUL5 binds to elongin BC complex, CUL3 binds to BTB-domain proteins, and CUL4A binds to DNA-damage-binding protein-1 (DDB1) [70]. Substrate receptor proteins bridge the E3 ligase with specific substrate. For example, the CUL1-Skp1 or CUL7-Skp1 complex binds to substrate receptors that encodes an F-box motif, the CUL2-elongin BC or CUL5-elongin BC complex binds to those encoding a suppressor of cytokine signaling (SOCS) box motif, and the CUL3-BTB-domain complex binds to their substrates via the C-terminus. Many viral proteins bridges the cullin complex with their specific substrate proteins: e.g., paramyxovirus V proteins, human immunodeficiency virus Vif and Vpr proteins, adenovirus E1b55K and E4orf6 proteins [67, 72–78]. Therefore, unique specificity of virulence factors to substrate proteins may contribute to the pathogenesis of viruses in animal hosts.

**Fig. 2:**
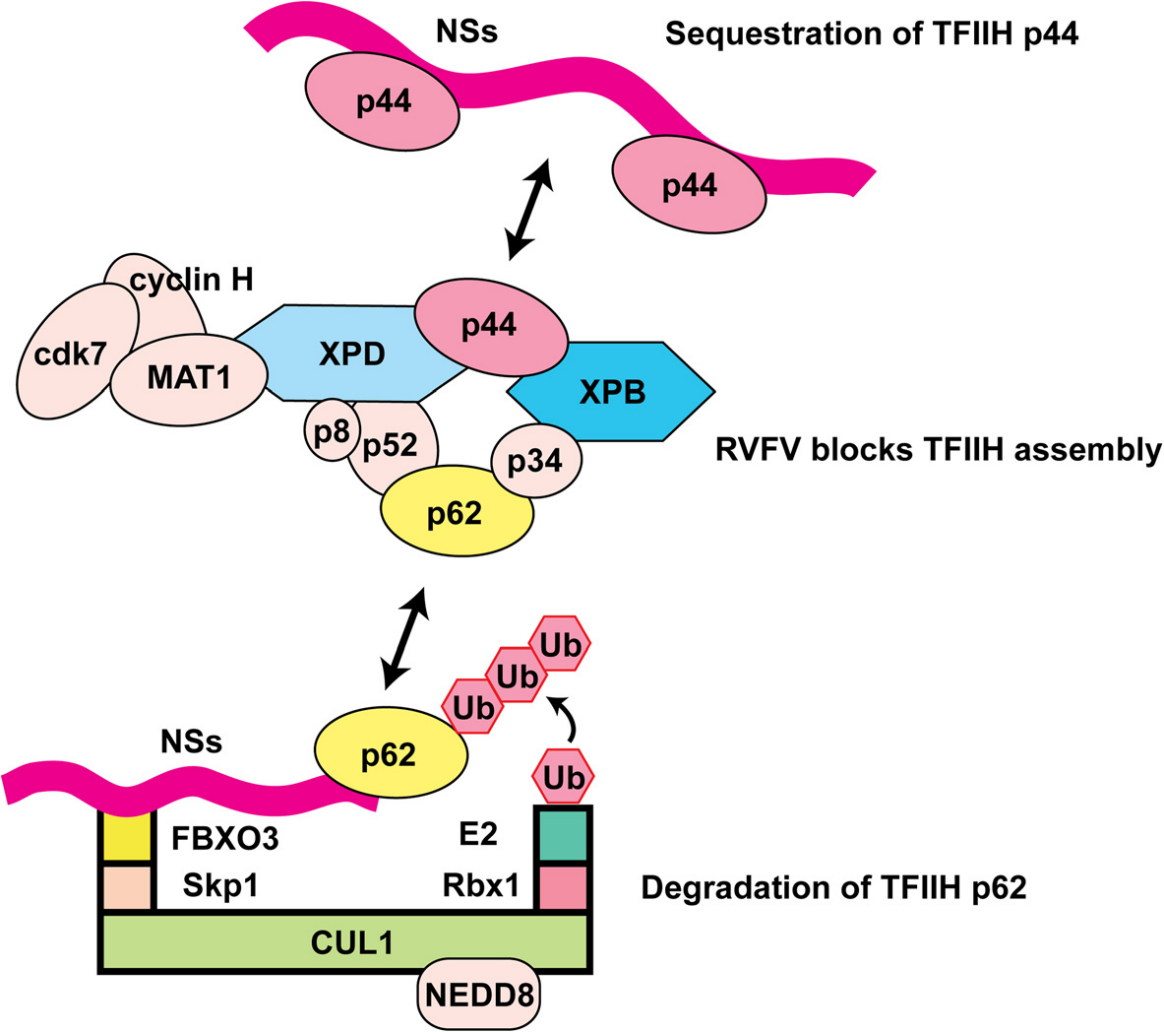
Schematics of RVFV NSs-mediated TFIIH suppression. The *top* portion of the figure illustrates that RVFV NSs protein binds to p44, and sequesters it from the assembly site of TFIIH. Whereas the *bottom* portion of the figure illustrates the formation of the E3 ligase complex, consisting of cullin 1 (CUL1), Skp1, and FBXO3, and promoting the subsequent degradation of p62 through RVFV NSs

To characterize the mechanism of TFIIH p62 protein degradation induced by RVFV NSs protein, proteomic screening using recombinant RVFV encoding NSs with a C-terminal TAP tag was accomplished. This resulted in the identification of the F-box protein (FBXO3) as an interacting partner protein for RVFV NSs protein [69]. Knockdown of FBXO3 or Skp1, inhibited p62 degradation from RVFV NSs protein, however knockdown of Rbx, CUL1 or CUL7 did not. The study also showed that the interaction of RVFV NSs protein with CUL1 most likely occurs through FBXO3 and Skp1 (Fig. 2). However, the ubiquitination of p62 could not be demonstrated. The FBXO3 (471 aa) has a full-length form (FBXO3/1) and a C-terminus truncated short form (FBXO3/2: 415 aa). Though FBXO3/1 is expressed in both the nucleus and cytoplasm, the amount of FBXO3/1 proteins, but not FBXO3/2 proteins, was shown to be decreased in cells infected with RVFV. This in turn indicates the alteration of protein turnover in FBXO3/1 proteins, which is due to its incorporation into the E3 ligase complex.

Unlike RVFV NSs proteins, TOSV, SFTSV, or UUKV NSs proteins localize in the cytoplasm only, and not the nucleus [79–83]. The localization of other phlebovirus NSs is currently unknown. The use of click chemistry, which covalently links fluorescent dye to nascent RNA, allows for the measurement of newly synthesized host RNA in infected cells [84]. Cells that were infected with RVFV, showed decreased incorporation of 5-ethynyluridine (5-EU), a nucleoside analog, into newly synthesized nascent host mRNA. Decreased 5-EU labeling also occurred in cells infected with recombinant RVFV MP-12 strain (rMP-12) encoding PTV NSs, in the place of MP-12 NSs [85]. However, this was not the case with cells infected with rMP-12 encoding SFSV NSs or TOSV NSs [85–87]. Though the mechanism has yet to be defined, PTV NSs proteins may decrease host transcriptional activities. Transcriptional regulations induced by phlebovirus NSs proteins, other than RVFV NSs protein, will need to be further characterized toward better understanding of NSs proteins.

Bunyamwera virus (BUNV), which is a member of the *genus Orthobunyavirus* (Bunyamwera serogroup), expresses NSs proteins (101 amino acids) from the +1 ORF that overlaps the N ORF. The NSs proteins of BUNV is also presented in both in the cytoplasm and nucleus [88]. The NSs proteins of BUNV target cellular RNA polymerase II, by inhibiting the phosphorylation of the C-terminal domain (CTD), YSPTSPS repeats, of which various serine residues are phosphorylated to regulate transcription [89]. Serine at the 2nd or 5th position plays a role in mRNA elongation and transcriptional initiation, respectively. BUNV NSs protein inhibits CTD serine phosphorylation at the 2nd, and not the 5th position, thus blocking the transition from transcriptional initiation to mRNA elongation. BUNV NSs protein also interacts with the mediator of RNA polymerase II transcription subunit 8 (MED8) protein, which regulates the transcriptional function of RNA polymerase II [90]. Intriguingly, RNA polymerase II protein level was shown to be decreased in BUNV infected cells, whereas this reduction did not occur when BUNV NSs protein lacked the MED8-binding domain [90]. Léonard et al. proposed that BUNV NSs protein mediates the degradation of RNA polymerase II through the E3 ligase complex, because the MED8 is also incorporated into the Rbx1-CUL2-elongin BC complex [90, 91]. However, the role of BUNV NSs protein or MED8 in the putative E3 ligase complex in the degradation of RNA polymerase II remains unknown. La Crosse virus (LACV), which is a member of the California serogroup of the genus *Orthobunyavirus*, also expresses NSs protein (92 amino acids). The NSs protein of LACV has a 38 % amino acid identity with BUNV NSs protein [92]. Although LACV NSs protein does not bind to MED8, it also induces the inhibition of cellular RNA polymerase II CTD serine phosphorylation at the 2nd, and not at the 5th position [93]. Furthermore, LACV NSs protein also induces selective degradation of the hyperphosphorylated IIO form of the RNA polymerase II RPB1 subunit via the proteasome pathway. This in turn does not directly have any effects with the hypophosphorylated IIA form [93]. The degradation of RPB1 subunit occurs in cells when the RNA polymerase II is stalled during elongation [94]. However, it remains unknown whether LACV NSs protein interacts with the E3 ligase complex to specifically target RPB1 or not.

## RVFV and TOSV NSs protein promotes posttranslational degradation of dsRNA-dependent protein kinase (PKR)

It is known that RVFV can replicate in the presence of host transcriptional shutoff induced by actinomycin D (ActD) [95]. To determine the role of RVFV NSs protein in the presence of host transcriptional shutoff, viral replication was characterized in cells treated with ActD. MP-12 replication was not decreased when infected Vero cells were treated with ActD. However, recombinant MP-12 encoding *Renilla* Luciferase (rLuc) in the place of NSs (rMP12-rLuc), showed more than a 2 log reduction in viral titer when infected Vero cells were treated with ActD [96]. This indicated that RVFV NSs protein is capable of promoting viral replication in the presence of host transcriptional shutoff. Eukaryotic initiation factor (eIF) 2α was shown to be highly phosphorylated in cells infected with rMP12-rLuc and treated with ActD, whereas eIF2α phosphorylation was minimal in cells infected with parental MP-12 and treated with ActD [96].

Subsequently, it was also demonstrated that RVFV NSs protein promotes the degradation of PKR [96, 97]. PKR (551 amino acids; human) is a serine-threonine protein kinase that is ubiquitously expressed in mammalian cells [98–100]; the majority of PKR is found in the cytoplasm and less than 20 % is found in the nucleus [100]. The degradation of PKR in RVFV-infected cells occurred in both the cytoplasm and nucleus [96]. Proteasomal inhibitors, MG132 or lactacystin, stabilized PKR in the presence of RVFV NSs protein. The accumulation of phosphorylated eIF2α did not occur in ActD treated cells infected with recombinant MP-12 encoding a dominant-negative PKR (PKR∆E7). Since eIF2α phosphorylation leads to translation initiation shutoff, RVFV NSs protein serves a role in facilitating efficient viral translation through blocking the PKR-mediated eIF2α phosphorylation.

Using reverse genetics, a number of NSs point mutants were subsequently screened for functional phenotypes. Among those that were screened, the R173A mutant did not promote PKR degradation, yet was able to still induce transcriptional shutoff and inhibit IFN-β mRNA upregulation [101]. Thus phosphorylation of eIF2α was induced in cells infected with RVFV expressing NSs R173A mutant without ActD treatment. Although the mechanism to induce eIF2α phosphorylation by RVFV NSs R173A mutant is unknown, the study showed that RVFV NSs mediated transcription suppression and IFN-β gene suppression occur independently from PKR degradation. The R173A mutant did not interact with PKR, thus indicating the need of the NSs-PKR interaction to trigger PKR degradation.

**Fig. 3:**
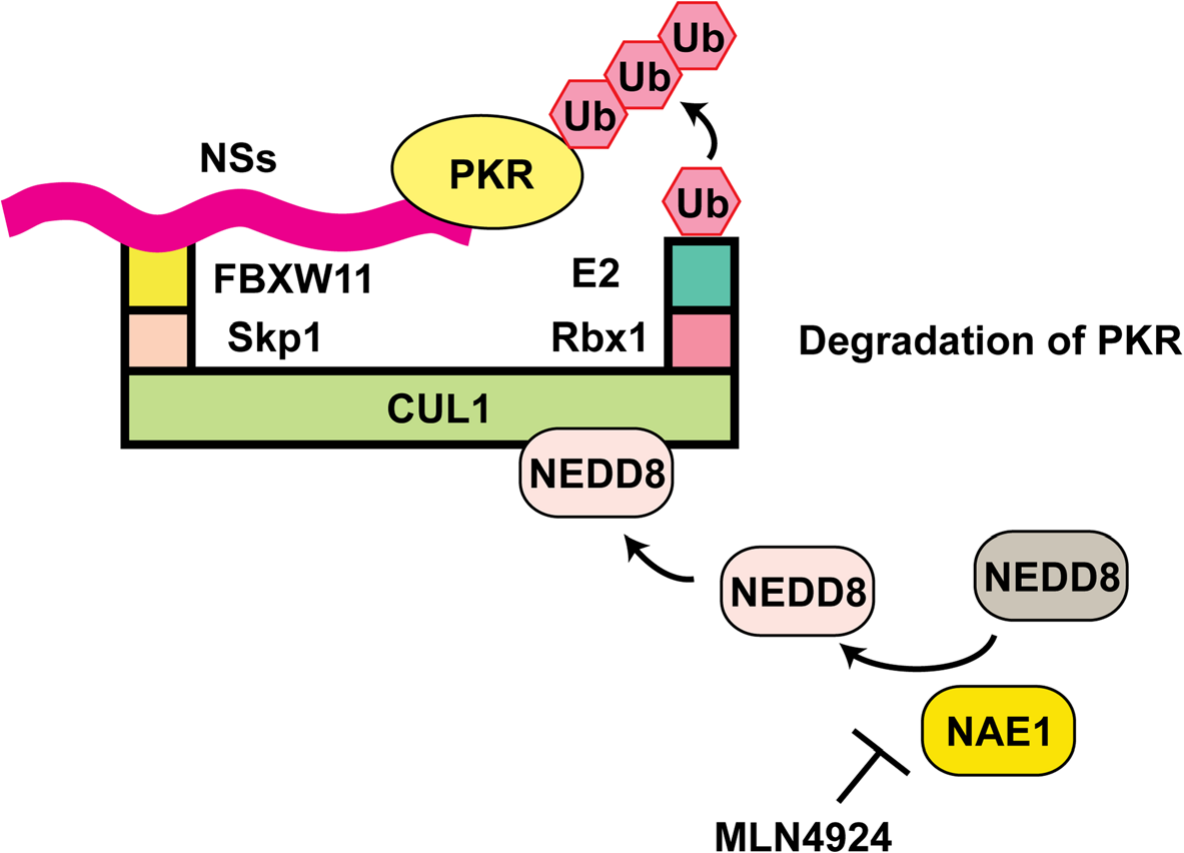
Schematics of RVFV NSs-mediated PKR degradation. RVFV NSs protein forms the E3 ligase complex, which consists of CUL1, Skp1, and FBXW11. The E3 ligase complex promotes the degradation of PKR via the ubiquitin-proteasome pathway. NEDD8 activating enzyme (NAE1) can be selectively inhibited by MLN4924

For NSs-mediated PKR degradation, the specific E3 ligase complex has been further characterized [102, 103] (Fig. 3). As described above, NEDD8 covalently binds to cullin (NEDDylation), to recruit E2 to E3 ligase. Thus the NEDD8 activating enzyme (NAE1) is required for the NEDDylation. As a small molecule inhibitor, MLN4924 functions as a potent and selective inhibitor for NAE1 [104]. As a result, in a dose-dependent manner, MLN4924 inhibited RVFV replication and increased the phosphorylation of PKR and eIF2α. RVFV NSs protein was also screened for potential interaction with CUL 1, 2, 3, 4A, 4B, 5, 7, or 9. Of which, RVFV NSs protein was found to only interact with CUL1. However, CUL1 mutants, ∆N53 or Y42A/M43A, which cannot interact with Skp1, was shown to be poorly bound to NSs protein. Therefore Skp1 mediates the interaction between NSs protein and CUL1. To identify specific F-box protein, which can interact with the CUL1-Skp1, 70 different F-box genes were screened via siRNA assay. As a result, the FBXW11 was screened out from siRNA pools from the reduction of RVFV replication [102] or PKR degradation [103]. Since BTRC (also known as βTrCP1) and FBXW11 (also known as βTrCP2) are functional paralogs of the *BTRC* gene (also known as *βTrCP* gene: Beta-transducin repeat containing protein), further protein analysis was subsequently carried out for both. The siRNA knockdown of both BTRC and FBXW11 mRNA inhibited RVFV replication significantly more than the single knockdown of FBXW11 [102]. Similarly, siRNA knockdown of BTRC gene expression in FBXW11 knockout cells also completely abolished PKR degradation by RVFV NSs protein [103]. The study also demonstrated that the C-terminal WD40 domain repeat of FBXW11 or BTRC, interacts with the C-terminal of NSs protein, which contains the “degron” sequence DDGFVE. Thus, RVFV NSs protein was identified as the adaptor protein connecting the CUL1-Skp1-FBXW11 or the CUL1-Skp1-BTRC complex with PKR. The ubiquitination of PKR could not be demonstrated, however, this may be due to low endogenous expression of PKR or high proteasomal activity.

TOSV NSs (316 aa.), but not that of PTV or SFSV, was also shown to promote the degradation of PKR similar to that of RVFV [87]. Though RVFV and TOSV share similar NSs functions, the amino acid sequence similarity is low, with no shared motifs identified. To better understand the potential mechanism commonly used in PKR degradation, it is important to characterize the role of TOSV NSs protein in the putative E3 ligase complex. This may be accomplished through identifying PKR binding motifs, and the type of cullin-RING complex used. It is also important to recognize whether phleboviruses, other than RVFV and TOSV, encode functions to facilitate viral translation without affecting the PKR.

NSs proteins from RVFV, BUNV, and LACV localize in both the nucleus and cytoplasm, and can induce a strong shutoff of host protein synthesis in a NSs-dependent manner [96, 105, 106]. Tick-borne UUKV or SFTSV expresses cytoplasmic NSs and does not induce host translational shutoff [107–109]. Although host translation is inhibited, RVFV, BUNV, or LACV can maintain viral protein synthesis during infection. Most bunyavirus mRNAs are unique, because they lack the polyA tail at the 3′ terminus. The polyadenylate binding protein 1 (PABP1) binds to the polyA tail and the cap of mRNA via eIF4G, and mRNA forms a circular structure for efficient translation [110]. RVFV NSs protein did not directly bind to PABP1, however, PABP1 was sequestered to nuclear speckles in cells expressing RVFV NSs protein [111, 112]. Nuclear retention of PABP also occurred in BUNV-infected cells at late stage of infection, and it probably due to decreased host mRNA in cytoplasm during NSs-mediated transcriptional shutoff [113]. Thus, RVFV and BUNV NSs proteins facilitate selective inhibition of host protein synthesis through transcription shutoff. The knockdown of eIF4G, but not PABP, decreased the translation of reporter gene flanked by BUNV 5′-UTR and 3′-UTR, which lacks a polyA tail [113]. Hantaviruses, on the other hand, do not induce host translational shutoff and some do not even encode the NSs gene. The N protein of hantaviruses functions as a surrogate for the eIF4F complex (eIF4A, eIF4G, and eIF4F), and thus recruits capped mRNA from the host to initiate viral translation [114]. Further study will be required to understand bunyavirus NSs functions regulating host and viral protein synthesis.

## Suppression of interferon (IFN)-β gene up-regulation by NSs protein of RVFV and other bunyaviruses

Type-I, II, and III interferons (IFNs) play important roles in the host defense against pathogens. IFNs exert their biological functions through the stimulation of target cells via specific IFN receptors, using autocrine and paracrine signaling processes. Through type-I IFN receptors (IFNAR), Type-I IFNs stimulate various types of cells, including lymphocytes, myeloid cells, epithelial cells, and stromal cells. Type-III IFNs show limited target specificity, such as epithelial cells, through type-III IFN receptor (IFNλR) [115–117]. Type-II IFN is dominantly expressed from T cells and NK cells, thus playing major roles in immunomodulation and antiviral activities through lymphocytes [118]. Type-I IFNs plays a major role in innate immunity during viral infections. Cells stimulated through IFNAR induce various IFN-stimulated genes (ISGs) to repress viral replication. IFNB gene promoter activation is induced via the coordinated binding of the homodimer of interferon regulatory factor 3 (IRF3) (or the heterodimer of IRF3 and IRF7), nuclear translocation of NFkB, and ATF2/cJun (AP1) to the positive regulatory *cis*-elements for IFNB gene [119]. The activation of these transcription factors are triggered by the recognition of replicating viral RNA from pattern recognition receptors (PRRs), such as retinoic acid-inducible gene I (RIG-I), melanoma differentiation-associated gene 5 (MDA5), or endosomal viral RNA through Toll-like receptors (TLRs) [115, 119]. Binding of viral RNA to RIG-I or MDA5 leads to their interaction with mitochondrial antiviral signaling protein (MAVS), and activation of TBK1 [tumor necrosis-receptor associated factor (TRAF) family member associated NFkB activator-binding kinase 1] [119]. Activated TBK1 induces phosphorylation and dimerization of IRF3 and IRF7.

RVFV NSs protein is a type-I IFN antagonist, and thus supports efficient viral replication in type-I IFN-competent cells or immune-competent hosts [120, 121]. A natural isolate, RVFV Clone 13 strain (C13), encodes an in-frame truncation of the NSs ORF from aa.16 to 198 (69 % of NSs ORF). The IFN-β mRNA can be upregulated as early as 3–6 h post infection with the C13 strain [60]. Bouloy et al. analyzed the virulence of reassortant RVFV generated between C13 and ZH548 strains in inbred 129/SvPaslco mice. They confirmed the attenuation of RVFV encoding C13 S-segment, and the rapid induction of type-I IFNs in the absence of intact NSs [121]. Therefore, the NSs is considered to be the major virulence factor for RVFV. Cells infected with RVFV strain encoding intact NSs protein did not upregulate IFN-β mRNA, but still activated RIG-I, dimerization and nuclear translocation of IRF3, and nuclear translocation of NFkB and AP1 [60, 122]. Thus, RVFV NSs protein inhibits the upregulation of IFN-β promoter downstream of the activation of transcription factors. Le May et al. [123] demonstrated that NSs protein binds to Sin3A Associated Protein 30 (SAP30) and subsequently prevents the activation of IFN-β promoter via interaction with a transcription factor, Yin Yang 1 (YY1) protein in mouse cells [124]. In the presence of NSs-SAP30-YY1 complex, the CREB-binding protein was not recruited to the IFN-β promoter, and subsequent acetylation of histone K8H4 or K14H3 did not occur. The RVFV NSs protein aa.210-230 was mapped to bind to SAP30 protein, and replication of recombinant RVFV encoding NSs∆210-230 induced IFN-β mRNA in infected cells. In addition, the NSs∆210-230 protein did not form NSs filament, or interact with SAP30 or IFN-β promoter. Thus, this study indicated that IFN-β promoter activation is inhibited from the interaction between RVFV NSs protein and SAP30 protein.

On the other hand, 11 different RVFV NSs mutants, encoding serial in-frame 25 aa truncation, did not suppress IFN-β gene upregulation, regardless of the location of truncations [125]. This indicated that RVFV NSs mutants failed to exert biological functions due to structural changes or alteration of subcellular localizations. Another study demonstrated that the TFIIH inhibition may also be responsible for the IFN-β promoter suppression. The siRNA knockdown of FBXO3 mRNA (E3 ligase for p62) allowed induction of IFN-β mRNA during RVFV infection [69]. The gaps in biological phenotypes should be further investigated for a better understanding of the mechanism.

TOSV NSs protein localizes to the cytoplasm, and inhibits nuclear localization of IRF3 [79]. In addition, TOSV NSs protein interacted with the N-terminal CARD domain of RIG-I, and promoted the degradation of RIG-I via the proteasome [126]. The inhibition of IFN-β clearly occurred when TOSV NSs was expressed from plasmid DNA or recombinant RVFV, however, it was weak in cells infected with authentic TOSV. This may most likely be due to the low expression level of NSs [79, 86, 126]. Later on, it was suggested that TOSV isolates from the Spanish lineage inhibits IFN-β gene more efficiently than those from the Italian lineage [127].

SFSV NSs protein inhibits the induction of IFN-β gene, however, the inhibition mechanism of type-I IFN suppression remains unknown [85, 97]. In addition, SFSV NSs protein induces neither host general transcription shutoff, nor PKR degradation [85, 97]. However, cells transfected with a reporter plasmid expressing *Renilla* luciferase under constitutively active SV40 promoter, showed an increase in reporter activity when SFSV NSs proteins were co-expressed [85]. Thus, SFSV NSs protein probably encodes a biological function upregulating host gene expression either via transcriptional or translational level, with unknown mechanism.

PTV NSs protein also inhibits IFN-β gene induction. The PTV *Adames* strain is highly pathogenic in hamsters and mice, whereas the PTV *Balliet* strain does not [128–130]. Similar to TOSV infection, authentic PTV did not completely inhibit the induction of IFN-β in infected cells. When infected with the PTV *Balliet* strain, type-I IFN was induced more abundantly in hamster embryonic fibroblast cells or mouse primary macrophages than with the *Adames* strain [130, 131]. In addition, PTV NSs protein expressed from plasmids or recombinant RVFV exerted a more efficient biological function than that expressed from PTV. In a reporter assay, PTV *Balliet* strain NSs protein inhibited Sendai virus-mediated IFN-β promoter activation less efficiently than PTV *Adames* strain NSs protein (~4-fold) [130]. Recombinant RVFV MP-12 strain encoding PTV *Adames* strain NSs was able to completely inhibit the up-regulation of mouse IFN-β mRNA and ISG56 mRNA [85]. It remains unknown how PTV NSs protein targets type-I IFN induction pathway, or how the replications of PTV *Adames* and PTV *Balliet* strains differentially induce type-I IFNs in the presence of NSs protein.

Tick-borne SFTSV NSs protein forms cytoplasmic inclusion bodies, binds to IRF3 via TBK1 (or IKKε) and then relocates them to cytoplasmic inclusion bodies. This in turn inhibits the induction of IFN-β gene, since IRF3 is being sequestered to cytoplasmic inclusion bodies [82, 83, 132]. In inclusion bodies, the SFTSV NSs-TBK1 complex was also associated with RIG-I and the E3 ligase TRIM25 [83]. Interestingly, SFTSV NSs inclusion bodies sequestered the cellular signal transducer and activator of transcription 2 (STAT2) and relocated both STAT1 and STAT2 to cytoplasmic inclusion bodies [133]. Thus, SFTSV NSs inclusion bodies play a key role in the inhibition of type-I IFN induction and signaling pathways. Other studies also indicated that SFTSV NSs protein forms viroplasm-like structure, containing NSs, N, dsRNA, and lipid droplets, suggesting the role of SFTSV NSs as a scaffold protein for viral RNA replication [81].

BUNV NSs protein inhibits IFN-β gene upregulation [105, 134] but does not inhibit the homodimerization or nuclear translocation of IRF3 [135]. Thus, the suppression of IFN-β promoter by BUNV NSs occurs downstream of the IRF3 activation. BUNV NSs protein may also inhibit IFN-β gene upregulation directly via blocking mRNA elongation of host RNA polymerase II [89].

Similarly to the IFN-β promoter suppression mechanism through SAP30-NSs binding, RVFV NSs protein also alters the process of mitosis in infected cells. RVFV-infected cells showed abnormal nuclei, such as lobulated nuclei, intranuclear DNA bridge, or micronuclei, in an NSs-dependent manner [136]. Through the SAP30-binding domain (amino acid 210–230), RVFV NSs filament interacted with pericentromeric major γ-satellite sequence, but not centromeric minor α-satellite sequence. It was also indicated that YY1 may mediate the interaction between the NSs-SAP30 complex and the γ-satellite sequence DNA. Since RVFV NSs protein accumulates in the nucleus, host cells also induce DNA damage signaling, including ataxia-telangiectasia mutated (ATM), checkpoint kinase 2 (Chk.2), and p53 [137]. RVFV NSs protein also triggers cell cycle arrests, either at the S phase (MP-12 strain) or the G0/G1 phase (ZH548 strain) [137]. Abnormal replication of infected cells, through NSs-mediated DNA damages, affects normal tissue differentiation, and may play a part in the pathogenesis of fetal malformation in infected ruminants.

Although RVFV NSs proteins shutoff host general transcription, including IFN-β gene, the host immune response to RVFV infected cells in vivo is unpredictable. Cytokine profiles were analyzed from human sera: six fatal and 20 non-fatal cases in Saudi Arabia [138], or 19 fatal and 85 non-fatal cases in South Africa [139]. Fatal cases showed elevated IL-10 (suppression of cell-mediated immune responses), IL-1RA (antagonist for IL-1), IP-10, MCP-1, CXCL9, or IL-8, indicating that fatal cases of RVF failed to mount a systemic pro-inflammatory cytokine response. Mitochondrial antiviral signaling (*Mavs*) gene knockout mice are a highly lethal RVFV infection model using MP-12 strain, and MCP-1, IL-10, IL-6, and CXCL9 were also significantly increased during RVFV infection [122]. The transcriptional profile of RVFV infected mouse cells was characterized at a relatively early stage of infection (8 hpi) using Chromatin immunoprecipitation (ChIP) and a mouse promoter array [140]. RVFV ZH548 strain showed significant downregulation of ten genes, including *Fbox3*, *Mapk8ip3*, *Stat2*, *IL3*, *IL10rb*, *Tyk2*, *Casp9*, *Phf21*, *Ncoa3*, and *Notch4*, when compared to recombinant ZH548 lacking NSs (ZH∆NSs). This indicated the impact of NSs expression on apoptosis, innate immunity, and other immunological pathways. Accordingly, cytokine profiles can be important markers to predict the severity of RVFV infection, and will thus be useful to evaluate the effect of antiviral treatments.

## Conclusion

Genetically, bunyaviruses are highly diverse, and some species can cause lethal infections in humans or livestock. RVFV is one of the most important bunyaviruses, and has caused large outbreaks in both ruminants and humans, resulting in devastating economic loss in affected regions. The major virulence factor NSs, exerts several different biological functions to hijack infected cells. Each mechanism is apparently similar to those used by other bunyavirus NSs proteins, as summarized in Table 1. The degradation of PKR is induced by RVFV and TOSV NSs proteins. RVFV NSs protein also promotes the degradation of TFIIH p62 protein, whereas TOSV NSs protein triggers the degradation of RIG-I. Since RVFV NSs protein functions as viral adaptor protein to form the E3 ligase complex for p62 or PKR degradation, similar mechanisms may be utilized by TOSV NSs protein for PKR or RIG-I degradation. Host general transcription suppression is induced by RVFV, BUNV, and LACV NSs proteins. These NSs proteins induce DNA damage responses, and trigger host translational shutoff. The expression of IFN-β gene is suppressed at the transcriptional level, which is in sharp contrast with other NSs proteins, as they only localize in the cytoplasm (e.g., TOSV, SFTSV). TOSV or SFTSV NSs proteins inhibit specific proteins in the IFN-β gene induction pathway (e.g., RIG-I, TBK-1). Further grouping of phleboviruses and orthobunyaviruses based on NSs protein functions will lead to effective strategies geared towards combating pathogenic bunyaviruses. Many bunyaviruses are neglected from funding agencies or public health policies, due to restricted viral distributions in specific arthropod vectors (e.g., sandfly, ticks), as well as limited marketability of vaccines or antivirals. Thus, further understanding of the unique biological function of the NSs protein in viruses that are currently uncharacterized, will be important in uncovering viral strategies used to adapt to both arthropod insects and vertebrates.

**Table 1:** NSs functions of RVFV and other bunyaviruses

Species	NSs localization	NSs functions	Proposed mechanisms
RVFV	N, C	Host general transcription suppression	Sequestration of TFIIH p44 [62]
			Degradation of TFIIH p62 [64, 69]
		Suppression of IFN-β gene activation	Interaction with SAP30 [123]
		Facilitation of viral translation	Degradation of PKR [96, 97, 102, 103]
			Sequestration of PABP1 [111, 112]
TOSV	C	Suppression of IFN-β gene activation	Degradation of RIG-I [79, 126]
		Facilitation of viral translation	Degradation of PKR [87]
SFTSV	C	Suppression of IFN-β gene activation	Sequestration of TBK1/IKKε [82, 83, 132]
		Suppression of IFN-β signaling	Sequestration of STAT2 [133]
		Facilitation of viral replication	Virosome-like structure [81]
BUNV	N, C	Host general transcription suppression	Inhibition of RNA pol-II [89, 90]
		Suppression of IFN-β gene activation	Inhibition of RNA pol-II? [135]
		Facilitation of viral translation	Unknown [105]
LACV	N, C	Host general transcription suppression	Inhibition of RNA pol-II [93]
		Suppression of IFN-β gene activation	Inhibition of RNA pol-II? [93]
		Facilitation of viral translation	Unknown [106]

*N* nucleus, *C* cytoplasm

## Abbreviations

ActD, actinomycin D; AP1, activating transcription factor 2/cJun; APOBEC3G, the apolipoprotein B mRNA editing enzyme catalytic polypeptide 3G; ATM, ataxia-telangiectasia mutated; ATR, ataxia-telangiectasia mutated and Rad3-related kinase; BC, elongin BC; BTB, broad-complex, tramtrack and bric a brac; BTRC, beta-transducin repeat containing E3 ubiquitin protein kinase; BUNV, bunyamwera virus; Cdk7, cyclin-dependent kinase 7; ChIP, chromatin immunoprecipitation; Chk.2, checkpoint kinase 2; CTD, C-terminal domain; CUL, cullin; CXCL9, chemokine (C-X-C motif) ligand 9; dsRBMs, dsRNA-binding motifs; eIF2α, eukaryotic initiation factor 2α; eIF4F, eukaryotic initiation factor 4 F; eIF4G, eukaryotic initiation factor 4G; FRIV, Frijoles virus; HIV, human immunodeficiency virus; ICOV, Icoaraci virus; IFN, interferon; IFNAR, interferon-α/β receptor; IRF3, interferon regulatory factor 3; IRF7, interferon regulatory factor 7; ISGs, IFN-stimulated genes; LACV, La Crosse virus; L-segment, large-segment; MAT1, menage a trois 1; MAVS, mitochondrial antiviral signaling; MCP-1, monocyte chemoattractant protein-1; MDA5, melanoma differentiation-associated gene 5; M-segment, medium-segment; NAE1, NEDD8 activating enzyme 1; NEDD8, neural precursor cell expressed developmentally downregulated protein 8; NIAID, National Institute of Allergy and Infectious Diseases; NIH, National Institutes of Health; NS, nonstructural protein; NSs, nonstructural protein on the S segment; ORF, open reading frame; PABP1, polyadenylate binding protein 1; PKR, dsRNA-dependent protein kinase; PRRs, pattern recognition receptors; PTV, Punta Toro virus; RIG-I, retinoic acid-inducible gene 1; RING, really interesting new gene; rLuc, *Renilla* luciferase; RVF, Rift Valley fever; RVFV, Rift Valley fever virus; SAP30, Sin3A-associated 30-kD protein; SFSV, sandfly fever sicilian virus; SFTS, severe fever with thrombocytopenia syndrome; SFTSV, severe fever with thrombocytopenia syndrome virus; Skp1, S-phase kinase-associated protein 1; SOCS, suppressor of cytokine signaling; S-segment, small-segment; STATs, signal transducers and activators of transcription; TBP, TATA-binding protein; TFIIH, transcription factor IIH; TLRs, toll-like receptors; TOSV, toscana virus; TRAF, tumor necrosis-receptor associated factor; Ub, ubiquitin; UUKV, uukuniemi virus; XPB, xeroderma pigmentosum type B; XPD, xeroderma pigmentosum group D; YY1, Yin Yang 1; βTrCP, Beta-transducin repeat containing protein
